# The Crusade against Abortionists

**Published:** 1911-11

**Authors:** 


### BUFFALO MEDICAL JOURNAL

**A Monthly Review of Medicine and Surgery**

*EDITOR*

A. L. BENEDICT.

**All communications, whether of a literary or business nature, books for review and exchanges, should be addressed to the editor, 354 Franklin Street, Buffalo, N. Y Please make personal or telephonic calls before 1 p. m.**

**The Buffalo Medical Journal will publish regularly, full transactions of any professional organization in western New York at cost. Personal notices, brief reports of society proceedings, and the like, are always welcome and will be published without charge.**

**Subscriptions may commence at any time. $2.00 per year in advance,**

Vol. LXVII. NOVEMBER, 1911. No. 4

### The Crusade Against Abortionists

IT is from no neglect of medical news that we have ignored various press items on this subject. We feel that, until definite judicial decisions have been reached, it would be an injustice to the physicians charged with the crime of abortion or its attempt, to bring them into further notoriety. We can neither defend nor condemn until all the evidence has been weighed bv an impartial court and a legal decision reached. Merely to mention a charge, may bring into question the reputation of an innocent man, among his professional brethren at a distance, who may fail to see the final notice of his acquittal.

We have much sympathy with a few members of the profession, who feel that their attempt to make prospective parents realize that abortion is murder, has been wasted on hired detectives. We can appreciate their indignation but, on the other hand, they as well as others should realize that the officials of the Medical Society of the County of Erie, are dealing with a vital problem and are trying to prevent a crime whose detection in genuine cases, is well nigh impossible. In this attempt, they must use such measures as afforded a reasonable chance of securing convictions, and must employ such agents as are available. It is better to detect promptly the man who is willing to perform a criminal abortion and to punish him on the lighter charge of intent and attempt, than to wait till he has destroyed dozens, perhaps hundreds of human lives in embryo and to convict him when, to these crimes, he has added death or serious illness of an adult woman.

We also agree most heartily with the opinion voiced by some critics, that the committee should "hew to the line, let the chips fall where they will." We do not agree with the insinuation that the men who are bearing the brunt of the labor of this thankless but very necessary task, are actuated by personal spite or are deliberately ignoring clues to influential, pious and respectable hypocrites. If any critic has good reason to believe that men of prominence in the profession are engaged in the abortion business, let him make his charges in such a way as to lead to action.

There is one further fact that must not be forgotten. An abortion, spontaneous or started in whatever way and by whatever means, is a serious medico-surgical case, requiring prompt attention by a skilled physician and having the utmost claim to professional secrecy. This should be borne in mind, both to secure an impartial attitude toward the men accused of crime and lest in the fear of a trap, other men refuse the assistance which humanity demands. But we would call attention to a well established ethical principle: that, except in the gravest emergency, foetal life should not be sacrificed without council. When for any reason; this is impossible, it would be a wise precaution to lay the facts before some reputable confrere as soon as possible, and to secure his collaboration and sharing of responsibility in the further conduct of the case.

